# Progressing the construct of enjoyment: conceptualizing enjoyment as a proactive process

**DOI:** 10.1007/s44202-021-00015-1

**Published:** 2022-01-04

**Authors:** Masato Kawabata, Clifford J. Mallett

**Affiliations:** 1 National Institute of Education, Nanyang Technological University, 1 Nanyang Walk, Singapore, 637616 Singapore; 2 School of Human Movement and Nutrition Sciences, The University of Queensland, Brisbane, QLD 4072 Australia; 3 Department of Sport and Health Sciences, Technical University of Munich, Uptown Munich, 80992 Munich, Germany

**Keywords:** Enjoy, Emotion, Hedonia, Eudaimonia, Proactive, Review

## Abstract

Enjoyment is an important psychological construct in many life domains. Despite the importance of the construct, conceptual clarity in what enjoyment is remains elusive. The elusive understanding of enjoyment is probably caused by conceptual ambiguity of the construct and a confusion in the public usage between hedonic and eudaimonic qualities of positive feelings. The hedonic quality of positive feelings (e.g., fun) reflects the simple attainment of desires; whereas the eudaimonic quality of positive feelings (e.g., joy) reflects fulfilling or realizing one’s true nature through full functioning of one’s ability. To better understand this important construct of enjoyment, we conducted a focused review of relevant literature. In the first section, relevant literature was reviewed to identify conceptual ambiguities contributing to why enjoyment has remained an elusive construct in research. In the second section, an operational conceptualization of enjoyment was proposed from integrative perspectives to overcome the identified issues. We proposed operationally conceptualizing enjoyment as *a proactive behavioral and psychological process towards the eudaimonic or hedonic qualities of positive feelings*. In this process, the individual appraises the situation in a positive way and commits oneself to savoring the situation and engaging in the task to have positive feelings of joy and fun. We explained why the operational conceptualization is important and useful from theoretical, empirical, and practical perspectives. In doing so, we also proposed possible future research directions with the operational conceptualization of enjoyment.

## Introduction

*Enjoyment* is an important psychological construct in many life domains, including sport and exercise. Indeed, a search of psychological literature based on the PsychINFO database revealed that 640 articles with the keyword of enjoyment in their title were published in peer-reviewed journals for the last 10 years (from January 2010 to December 2021), and 124 of 640 articles (19.4%) were related to sport and exercise. Enjoyment is one of the most important predictors of sport commitment and participation in both youth and elite sport [1]. Furthermore, Biddle et al. [2] stated that enjoyment is critical in some structured exercise activities where physical effort is required. In spite of the importance of the construct to participants in sport and exercise settings, conceptual clarity in what enjoyment is remains elusive [2]. The elusive understanding of enjoyment is probably caused by an unclear definition of the construct and use of the term interchangeably in relation to both hedonic quality of positive feelings (e.g., comfort, easy, effortless, fun, pleasure) and eudaimonic quality of positive feelings (e.g., joy, achieving, elevating, fulfilling, meaningful). To better understand the vital construct of enjoyment, it is important to have a clearer operational definition by differentiating hedonic and eudaimonic qualities of positive feelings based on the relevant literature.

Kapsner [3] wrote a brief section on enjoyment in the encyclopedia of positive psychology but cited only two references. They were rebuttal articles on enjoyment [4, 5], published in the *Journal of Sport & Exercise Psychology* more than two decades ago. In the review, Kapsner [3] stated that enjoyment is a key construct in many areas of research, but it remains unclear whether enjoyment is an affect, an experience, a cognitive perception, or some of combination of those, and therefore he recommended developing a widely accepted conceptual definition of enjoyment.

In the present study, we accepted this challenge to advance the research on the construct of enjoyment. In the first section, we reviewed relevant literature to clarify conceptual ambiguities contributing to this elusive nature of what is enjoyment. In the second section, we proposed an operational conceptualization of enjoyment from integrative perspectives to overcome the identified issues and understand this important construct better. Sport and exercise psychology is one of the prominent domains in which the construct of enjoyment has been actively investigated; therefore, we use sport as an example context to elucidate conceptual clarity.

## Why has enjoyment remained an elusive construct in research?

### Emotion, mood, and affect

One key conceptual issue is the ambiguity of whether enjoyment is an affect, an experience, a cognitive perception, or some combination of those [3]. To address this conceptual issue, it is necessary to understand the framework of key constructs such as emotion, mood, and affect that are relevant to the construct of enjoyment [4, 5]. *Emotion* is a cognitively appraised reaction to an event or stimulus [1, 6]; in contrast, *mood* is considered a more enduring state and less intense with less-specific reference objects [1, 7]. *Affect* is considered as a broader term or more general concept, including emotions and moods [1, 7, 8]. Emotions are often conceptualized as discrete categories of emotion families [6]; whereas affect is frequently conceptualized as changing along two dimensions, either pleasantness and activation dimensions [9] or positive and negative emotional activation dimensions [10].

Fredrickson [6] and Perkun et al. [7] conceptualize emotions as processes of multi-component/faceted phenomena. Fredrickson [6] stated that emotions are “best conceptualized as multicomponent response tendencies that unfold over relatively short time spans” (p. 218), and the response tendencies consist of the appraisal process, subjective experience, facial expression, cognitive processing, and physiological changes. Perkun et al. [7] stated that “emotions are multifaceted phenomena that involve several interrelated psychological processes” (p. 1), which contain subjective feelings (affective component of emotion), cognitions (cognitive component), motivational tendencies (motivational component), physiological processes (physiological component), and expressive behavior (expressive component). Shuman and Scherer [11] consider that the affective component is a necessary and core element of emotion (e.g., “I am afraid of competing in front of a large number of spectators”); whereas other emotion components (e.g., motivational component — “I want to escape from here”) may or may not be present when the emotional process is initiated. Although Perkun and colleagues [7] viewed enjoyment as one of the ‘activity emotions’ under ‘achievement emotions’, we advise some caution in viewing enjoyment as an emotion due to some key conceptual issues listed in the section below.

### Enjoyment, fun, joy, and pleasure

The word ‘enjoy’ is comprised of the prefix of ‘en-’ and the word of ‘joy’. The prefix of ‘en-’ means to “make someone or something be in a particular state or have a particular quality” [12]. Literally speaking, the meaning of ‘*en*-joy’ is to have joy and ‘*en*-joy-ment’ is the act of having joy.

What is joy then? Robbins [13] stated that “Joy is a pleasant and often quite intense emotion which usually occurs within a safe and secure environment and is experienced bodily as a warm glow which emerges from the center of the body and moves upward and outward” (p. 540). In *the Journal of Positive Psychology’s* special issue on joy, Emmons [14] stated that the systematic study of joy has been largely neglected in psychological science although joy is one of basic or primary human emotions and important to well-being. He considered that difficulty in defining joy may be one of reasons for the absence of systematic research on joy as some definitions were too vague and cannot be differentiated from other positive emotions (e.g., Lazarus [15] often used joy interchangeably with happiness). However, there was a general agreement among contributors to the special issue that joy is a positive emotional state and a positive affective response to some good object [14, 16, 17]. Emmons [14] stated that joy is associated with something larger than ourselves in a richer, more exuberant life. Watkins [17] suggested that joy happens when individuals connect or unite with someone or something important to them.

An oft-used term in sport is *fun* [18], especially in the youth sport literature [19–21]. Based on the consistently high correlation between fun and positive mood states scores, Wankel and Sefton [21] considered fun as a positive emotional state. Jackson [18] considered that the terms of fun and enjoyment are similar enough to be used interchangeably. Wankel and colleagues [21, 22] also used the two terms interchangeably. In contrast, Scanlan and colleagues [19, 23, 24] considered that fun is one of generalized feelings (e.g., pleasure, liking, and fun) under *sport enjoymen*t. Although enjoyment and fun share conceptual similarity, we advise caution in using the two terms interchangeably to understand the terms better from psychological science perspectives.

Scanlan and colleagues [19, 23, 24] also reported *pleasure* as one of generalized feelings. However, pleasure has been defined narrowly as the affective response to satisfying homeostatic needs (i.e., physiological needs for food, rest, or sex etc.) [25]. Consistent with this definition, Csikszentmihalyi [26] distinguished pleasure (satisfaction of the homeostatic needs) and enjoyment (satisfaction of the growth-oriented needs). Although Wankel [5] acknowledged some benefit of Csikszentmihalyi’s distinctions between pleasure and enjoyment, he considered that such distinctions may be counterproductive in understanding sports and physical activity participation because sport participants do not make a distinction between pleasure and enjoyment and their account of enjoyment is not limited to the matching of perceived skills and challenges as Csikszentmihalyi [26] proposed in the flow theory.

## A conceptualization of enjoyment from integrative perspectives

### Enjoyment as process for the positive feelings of joy and fun

Both joy and fun are considered a positive emotional state, as reviewed earlier. In contrast, enjoyment is viewed more generally than a discrete emotion [19]. We support this perspective as ‘*en*-joy-ment’ is the act of having joy as mentioned earlier. Watkins [17] proposed that “not all en*joy*ment [italics added originally] is joy; not all we delight in represents an experience of joy” (p. 27) and questioned whether Johnson’s [16] example of the delight in a gladiator’s game is an example of joy. Alternatively, Fredrickson [27] stated that joy shares conceptual space with other relatively high-arousal positive emotions such as amusement, elation, and gladness, and proposed that joy produces the urge to play and to be playful in the broadest sense of the term. In sport contexts, such playful aspects of joy are commonly described as fun as reviewed earlier. In addition, joy is associated with something larger than ourselves and important to ourselves [14, 17]. We do not consider that fun and joy can be completely separated empirically as they share conceptual similarities. However, it would be beneficial to investigate how different words (e.g., fun and joy) are used to describe different aspects of positive feelings under the processes of enjoyment. To this end, it is necessary to provide conceptual framework of the operational differentiation between fun and joy and obtain empirical support for the conceptual differentiation. For instance, it would be useful to examine whether appraisals are different between joy and fun [28, 29].

Johnson [16] stated that cross-cultural work is especially important to understand the concept of joy better. In Japanese, a four-character phrase ‘kidoairaku (喜怒哀楽)’ is used to describe human’s various emotions, in which ‘ki (喜)’, ‘do (怒)’, ‘ai (哀)’, and ‘raku (楽)’ mean ‘joy,’ ‘anger’, ‘sadness’, and ‘fun’, respectively. The positive emotions of joy and fun are more clearly differentiated in Japanese, compared to English. We list expected similarities and differences between fun and joy in Table 1. According to two distinct Greek philosophies, *hedonism* reflects “the view that well-being consists of pleasure or happiness,” whereas *eudaimonism* reflects “the belief that well-being consists of fulfilling or realizing one’s daimon or true nature” ([30], p. 143). In the eudaimonic view, well-being consists of being fully functioning rather than simply attaining desires [30–32]. Given that eudaimonic and hedonic views of well-being are related but distinguishable [33], it would be useful to apply eudaimonic and hedonic views to understanding the processes of enjoyment and examine how people experience and appraise enjoyment of fully and optimally functioning rather than simply accomplishing personal desires.

**Table 1:** Expected similarities and differences between fun and joy

	Fun	Joy
Positive/Negative emotions	Positive	Positive
Activation level	High	High
Quality based on the Greek philosophies	Hedonic	Eudaimonic
Age group	Young	Matured
Difficult level	Low-Moderate	﻿Moderate-High
Effort level	Low-﻿Moderate	﻿Moderate-High
Stress level	Low-﻿Moderate	﻿Moderate-High
Challenge level	Low-﻿Moderate	﻿Moderate-High
Meaningful level	Low-﻿Moderate	﻿Moderate-High
Serious level	Low-﻿Moderate	﻿Moderate-High

The hedonic quality of positive feelings reflects the simple attainment of desires, whereas the eudaimonic quality of positive feelings reflects fulfilling or realizing one’s true nature through full functioning of one’s ability

Huta and colleagues investigated how hedonic (e.g., fun, pleasure) and eudaimonic (living life to one’s potential) motives change across adulthood (18–87 years old) [34] and in youth (7–18 years old) [35].^1^ LeFebvre and Huta [34] reported that hedonic pleasure motives decreased from 30 s onward for both genders, and Gentzler et al. [35] found that hedonic motives were higher in a sample of children aged 7–12 years compared to adolescents aged 14–18 years. Fun is an oft-used term in the youth sport literature [19–21], as stated earlier. Considering youth is a major cohort of sport participants across countries, hedonic quality of positive feelings (e.g., fun, pleasure) might be emphasized when youth talk about the situation where they enjoy sports. However, hedonic quality of positive feelings (e.g., fun, pleasure) might not be most important for middle-aged adults onwards according to the finding of LeFebvre and Huta’s [34] study. Thus, there is a need to better understand eudaimonic quality of positive feelings (e.g., joy, fulfilling) across different age groups when they enjoy activities.

As mentioned earlier, the literal meaning of ‘*en*-joy’ is to have joy. There is also a phrase of ‘enjoy oneself’ in English. This literal meaning and phrase implies that the processes contributing to positive feelings are proactive. To understand the construct of enjoyment better and progress its research from integrated perspectives, we propose operationally conceptualizing enjoyment as *a* *proactive behavioral and psychological process towards the eudaimonic or hedonic qualities of positive feelings*. In other words, in the process, the individual appraises the situation in a positive way (e.g., interpreting the situation as a challenge rather than a threat) and commits oneself to savoring the situation and engaging in the task to have positive feelings of joy and fun. We explain below why the operational conceptualization is important and useful from theoretical, empirical, and practical perspectives.

### Benefits of an operational conceptualization

#### From theoretical/conceptual perspectives

With the operational conceptualization of enjoyment, we are able to clarify the construct by connecting it well with established psychological theories. For instance, the conceptualization of enjoyment as ‘a proactive behavioral and psychological process’ indicates that individuals need to engage in the task or activity to have eudaimonic or hedonic qualities of positive feelings of joy and fun rather than just waiting for something fun or joy to happen or simply responding to an event or stimulus. From the perspective of approach-avoidance motivational distinctions [37], the proactive processes are associated with approach motivation rather than avoidance motivation.

By integrating the positive feelings of joy and fun, we can study the different qualities of positive feelings as eudaimonic and hedonic aspects of enjoyment. The quality of enjoyment would be different between situations under the same person, even though the person states she or he enjoyed the activity. For example, the positive feelings experienced by elite athletes in very high achievement situations (e.g., the final match at Olympic Games or the Grand Slam tournaments) are certainly different from the positive feelings they have when they play their sport for interacting with their fans at a charity game, despite the possibility that both situations are meaningful for the player. Naomi Osaka [38], a Japanese tennis player, pointed out the importance of enjoying the inestimable situation (e.g., the final of Australian Open) by understating the meaning and value of the situation, accepting and committing to the challenge in the situation, to perform to her best.

#### From empirical perspectives

Many studies have been conducted to investigate and understand athlete motivation in various sport settings. According to the research based on motivation theories (e.g., self-determination theory), key points about how to shape adaptive motivational regulations have been well documented [39]. For example, satisfaction of sport participants’ basic psychological needs under needs-supportive motivational climate promotes their enjoyment level [40]. However, if we ask participants retrospectively whether they enjoyed the activity, we are unlikely to know whether the quality of enjoyment is similar between individuals with different motivational profiles. For instance, the quality of enjoyment when people engage in the activity purely with intrinsic motivation is unlikely to be the same as the quality of enjoyment when people engage in the activity with high integrated or identified regulation. It could be possible to investigate such differences in the quality of enjoyment by differentiating eudaimonic and hedonic qualities of positive emotions in the activity.

As reviewed earlier, emotions are described as multifaceted consisting of subjective feelings, cognitions, motivational tendencies, physiological processes, and expressive behavior [6, 7]. Perkun et al. [7] also differentiate activity emotions (i.e., emotions during activities) from outcome emotions (i.e.., emotions related to outcomes after the activity). These multiple components and the distinction between activity and outcome emotions would be useful to investigate, including possible factors and approaches that are related to the proactive processes towards the positive feelings of joy and fun. For example, the factors and approaches that result in hedonic quality of positive emotions (e.g., fun, pleasure) may be different from those eudaimonic quality of positive emotions (e.g., joy, fulfilling). Perhaps, comfort, easy and effortless nature of the activity or situations may be emphasized to have hedonic quality of positive feelings. In contrast, people may emphasize efforts and emotional or behavioral regulations in challenging or stressful situations to have eudaimonic quality of positive feelings (e.g., joy, fulfilling). The factors or approaches for eudaimonic quality of positive emotions (e.g., joy, fulfilling) might be more related to something meaningful, prized, challenging or higher levels of satisfaction of basic psychological needs [41]; whereas the factors or approaches for hedonic quality of positive emotions (e.g., fun, pleasure) might be more associated with lower levels of basic psychological need satisfaction. Furthermore, the positive feelings of joy and fun in the action should be differentiated from the positive feelings of pride or achievement after the action (e.g., games, competitions) as activity emotions are likely affected by outcomes if activity emotions are measured retrospectively. These differentiations are not clear according to the existing measures in sport (e.g., the Physical Activity Enjoyment Scale [42]; the Sport Enjoyment subscale of the Sport Commitment Questionnaire-2 [24]; the Sport Emotions Questionnaire [43]). With the clearer operational conceptualization of enjoyment, we can hopefully develop instruments and approaches to measure enjoyment in detail from integrated perspectives.

Although both joy and fun are considered a positive emotional state, it would be challenging to empirically separate them into discrete emotional constructs (e.g., extracting two distinguishable factors through factor analysis on self-reported data) as they share similar conceptual space. Kawabata and Asakawa [44] recently investigated whether Japanese undergraduate students (*N* = 247) differentiated the feeling of fulfillment from the feeling of fun. Around half of the participants (*n* = 131, 53.0%) considered the two constructs as identical, whereas the other half (*n* = 112, 45.3%) viewed them as different. However, both groups perceived the situation in which they had felt fulfillment was more difficult and effortful than the situation in which they felt fun. This study indicates that the relevant terms of positive feelings (e.g., enjoyment, fun, joy, and pleasure) might be used interchangeably in the general public; however, they are also aware the difference in the quality between the positive feelings. Perhaps, a three-dimensional response surface approach [45] or the combination of discrete and dimensional approaches might be useful to measure and demonstrate the differentiations between joy and fun empirically.

#### From practical perspectives

When enjoyment is better understood with our operational conceptualization, it would likely (i) reduce misunderstanding of positive feelings of joy and fun, and (ii) to promote positive feelings of joy and fun in sports. For instance, around six decades ago when the first Tokyo Sumer Olympics were held in 1964, Japanese athletes were not allowed to smile in training and competitions because coaches believed that athletes were not fully committing themselves to the training or completion when they smile or show their teeth in their smiling face while they are playing their sports. Even today, when athletes say that they want to enjoy competitions such as Olympics or Paralympics, laypeople or journalists in Japan who have not seriously participated in sports criticize the athletes for their statement, assuming that the athletes want to have hedonic quality of positive feeling (e.g., comfort, easy, effortless, fun) although they participate in Olympics as representatives of Japan and supported financially with public funds to participate in the competitions. These misunderstandings of enjoyment should be avoided to boost positive experiences in sporting settings. To this end, the clearer operational conceptualization of enjoyment would be useful to facilitate a greater understanding of enjoyment and avoid misunderstandings of the vital construct.

A deeper understanding of enjoyment as proactive processes from multi-component perspectives is likely to promote individual’s knowledge and skills to enjoy oneself in sport and consequently enhance actual performance and experiences, including top high-performance athletes. For instance, repetitive practice of mundane or unpleasant tasks is often required to acquire complex motor skills or do rehabilitation programs. It might be not fun for athletes to work on the mundane or unpleasant tasks repetitively. However, it might be possible to have joy if they appraise the situation positively and internalize and endorse the meaning or value of the training. Naomi Osaka [38], a four-time Grand Slam single tennis champion, stated that a feeling of gratitude for playing tennis under the Covid-19 pandemic positively affected her performance at the final in the Australian Open 2021. A Japanese swimmer, Rikako Ikee [46], who is the junior-world record holder in the 50 m freestyle and butterfly and was diagnosed leukemia in 2019, also stated the significance of her appreciation in participation in the final of 100 m butterfly at the Japanese swimming championship after overcoming leukemia a year earlier. She won the race and was qualified for the 2020 Tokyo Olympics. These are good examples in feeling joy is possible in elite competitive situations and the mindset that leads to that joy is useful to produce high performance.

To understand athletes’ responses to competition, Jones et al. [47] proposed the theory of challenge and threat states in athletes (TCTSA) based on the biopsychosocial model of challenge and threat [48], the model of adaptive approaches to competition [49], and the control model of debilitate and facilitative competitive state anxiety [50]. To promote positive experiences in sports, it is important to investigate how athletes have the feeling of joy in competitive or stressful situations by using the framework of TCTSA and differentiating activity emotions from outcome emotions. For instance, athletes might have uncomfortable or unpleasant feelings (e.g., nervous, stressful) even though they attempt to appraise the competitive situation as challenge. They may not be aware of their internal feelings while they are fully functioning during their performance. In short-duration sports (e.g., 100 m sprint), athletes might have a joyful feeling only after the race. In contrast, tennis or golf players might have several occasions (e.g., before or after initiating their shot or in the break time) to savor a feeling of joy to a certain degree during a match.

A significant question emerges from practical perspectives. How can we develop appropriate mindsets and acquire skills and knowledge to enjoy oneself in adverse sport situations? It is also important to investigate whether the mindsets and skills to enjoy oneself can be enhanced regardless of a personal trait related to enjoying the task [51]. As mentioned earlier, key strategies for motivating other people positively have been well documented [39, 40]. However, can we develop such mindsets and skills on our own? How might other social actors (parents, coaches) contribute or thwart these adaptive mindsets? Conceptualizing enjoyment as a proactive process and investigating the process from multi-component perspectives [7] is considered useful to understanding the mechanisms of how to make uninteresting, boring, or stressful situations joyful or fun. Given that sport is an ideal situation to understand the effect of emotions on performance and the efficacy of strategies for emotions regulation [1], positive education programs to teach the mindsets and skills through sports would be possible and meaningful [52].﻿

## Conclusions

In the present study, we conducted focused reviews of relevant literature on enjoyment and clarified some conceptual issues that probably contributed to enjoyment remaining an elusive concept in research. We used sport as a specific context to elucidate conceptual clarity since the construct of enjoyment has been actively investigated in sport and sport is an ideal situation to understand the effect of emotions on performance [1, 53].

In reviewing the literature, several issues emerged. First, whether enjoyment should be viewed as an emotion. Another issue was that relevant terms (e.g., fun and enjoyment) have been used interchangeably. To overcome these issues, we proposed operationally conceptualizing enjoyment as *a* *proactive behavioral and psychological process towards the eudaimonic or hedonic qualities of positive feelings*. We explained the reasons why the operational conceptualization is useful from theoretical, empirical, and practical perspectives by using sport settings as examples.

We also proposed possible future research directions with the operational conceptualization of enjoyment. To better understand the proactive behavioral and psychological processes, it is considered useful to explore how the factors and approaches that result in hedonic quality of positive emotions is different from the eudaimonic quality of positive emotions by differentiating activity emotions from outcome emotions. Furthermore, for promoting positive experiences, it is important to investigate the mechanisms of how to make uninteresting, boring, or stressful situations joyful or fun that are guided by established theoretical frameworks.

The operational conceptualization of enjoyment we proposed in this paper could be used in other contexts such as education and clinical settings and the findings of possible future research in sport would be extended to other contexts. However, the operational conceptualization was proposed based on a focused review of the relevant literature and the context of our examples was limited to sport settings. Thus, the usefulness of our operational conceptualization of enjoyment should be rigorously examined in various contexts.

Enjoyment is an important psychological construct in many life domains. However, conceptual clarity in what enjoyment is, has remained elusive. In the present paper, we took on the challenge to advance research on the construct of enjoyment from integrative and cross-cultural perspectives. In conclusion, our operational definition of enjoyment would be useful to progress the research on enjoyment and better understand this vital construct. It is anticipated that the present paper can serve as a catalyst for future studies on enjoyment.

## Data Availability

Not applicable.

## Footnotes

1 Hedonic and eudaimonic motives were measured by the Hedonic and Eudaimonic Motives for Activities Scale [36] and the Hedonic and Eudaimonic and Extrinsic Motives for Activities Scale [34]. In the scales, the item of ‘seeking enjoyment’ is one of five items under the hedonic motive subscale.
